# The Dataset of Camellia Cultivars Names in the World

**DOI:** 10.3897/BDJ.9.e61646

**Published:** 2021-01-19

**Authors:** Yanan Wang, Huifu Zhuang, Yunguang Shen, Yuhua Wang, Zhonglang Wang

**Affiliations:** 1 Service Center for Information Technology, Kunming Institute of Botany, Chinese Academy of Sciences, Kunming, China Service Center for Information Technology, Kunming Institute of Botany, Chinese Academy of Sciences Kunming China; 2 Kunming Botanical Garden, Kunming Institute of Botany, Chinese Academy of Sciences, Kunming, China Kunming Botanical Garden, Kunming Institute of Botany, Chinese Academy of Sciences Kunming China; 3 Yunnan Key Laboratory for Wild Plant Resources, Kunming Institute of Botany, Chinese Academy of Sciences, Kunming, China Yunnan Key Laboratory for Wild Plant Resources, Kunming Institute of Botany, Chinese Academy of Sciences Kunming China

**Keywords:** Camellia, cultivar, synonym, registration, history

## Abstract

**Background:**

Camellias are popular ornamental, tea and woody-oil plants that have been cultivated throughout the world for centuries. To date, over 23,000 cultivars, with more than 45,000 cultivar names including synonyms, have been registered or published. A global digital dataset of Camellia names will provide a validated reference which can then serve to prevent further duplication when selecting names for new cultivars and assist in clearing up some of the confusion that still clings to a few of the old cultivar names. This comprehensive compendium is our review of camellia cultivars through history.

**New information:**

The camellia cultivar names were collected from books and journals, as well as new registrations. These were then reviewed by experts in the online working platform, the Database of International Camellia Register (DICR). After treating and correcting important issues that existed in camellia names, especially those with many re-used names and diacritical marks etc. in Japanese cultivars, a dataset of camellia names from sources throughout the world was summarised from the year 1253 to 2019. To date, a total of 45,210 cultivar names were released by different countries, including 23,887 accepted names and 21,323 synonyms. Excluding 3,944 names believed extinct, a total of 19,944 cultivar names are still in use. Amongst camellia cultivars, most (23,449) were for ornamental use, 429 were additionally used for tea and 228 for oil. *Camellia japonica* and its hybrids represent 18,141 or 74.10%, followed by those of *C. reticulata* and its hybrids (1,432 or 5.85%) and *C. sasanqua* and its hybrids (1,291 or 5.27%). The top five countries of origin are USA (7,502 cultivars), Japan (6,592), Italy (2,833), China (2,066) and Australia (1,216). The data showed the number of camellia cultivars per country is somewhat related to each country's economic prosperity. Managed by the International Camellia Registration team, this is the most comprehensive dataset in the genus *Camellia*. It will facilitate quick reference and scientific naming for breeders.

## Introduction

The genus *Camellia* arose in southeast Asia, with 80% of the species coming from China (Chang 1981, Gao et al. 2005). These include economically-important *C. sinensis* (Tea) and *C. oleifera* (oil), as well as decorative species, such as *C. japonica*, *C. reticulata* and *C. sasanqua* (Trehane 2007, Yi et al. 2015).

Camellia has a very long history of practical use, going back perhaps 5000 years for tea in China (Ming 2000, Pei and Huai 2007, Hamilton-Miller 1995). Dried tea spread westwards from China, playing an important role in international trade and cultural exchange over the great overland trade route, *the Silk Road*, to Persia (Iran) from the 8^th^ century and east across the sea to Japan (Trehane 2007, Yang and Yang 2019, Sharangi 2009, Saeed et al. 2017, Ding 2015). Dutch merchants were the first to bring actual tea leaves to Europe, to The Hague in 1610. Since the first seeds of *C. japonica* were introduced to Europe in 1620 to Portugal, ornamental camellias were then developed as their popularity spread worldwide (Jorge 2014, Chen et al. 2012). As camellia cultivation has a very long history, a great number of cultivars were released throughout the world.

In 1962, the International Camellia Society (ICS) was founded. It was then appointed as the International Registration Authority for the Genus *Camellia* at the International Horticultural Congress at Brussels. In 1993, *The International Camellia Register* (ICR) published a foremost piece of work consisting of two volumes with 2,208 pages (Savige 1993) with the first supplement in 1997 (Savige 1997). The second supplement was produced in 2011 (Haydon 2011). These four books together weighed 7.04 kg. The printed books were heavy to carry, so not easy to use. A digital dataset that included all camellia cultivars was urgently needed. During the last five years, our team checked ICR, carefully referencing the latest version of *The International Code of Nomenclature for Cultivated Plants* (ICNCP) (Brickell et al. 2016). We found important issues and problems existed in the printed ICR which needed treatment and solving as soon as possible. For example, the issue of duplicate names, especially in Japanese cultivars needed attention. After years of effort, we finally added all printed records of camellia names on to a digital database. In 2018, during the International Camellia Congress held in Nantes of France, we published the first Camellia Dictionary of Cultivars. In 2019, the Database of International Camellia Register (DICR, http://camellia.iflora.cn) was officially available for public use.

In this paper, a comprehensive dataset of world-wide camellia names has been summarised from the year 1253 to 2019, including all the names of camellia cultivars that appeared in books and journals, as well as new registrations from recent years. Those names have been reviewed by an international team in the DICR online working platform. The Ornamental, Tea and Oil description information in different languages including English, Chinese and Japanese was also collected. We treated all cultivar names to comply with the rules in ICNCP. To date, it is the most comprehensive dataset including all cultivars (Ornamental, Tea and Oil) in the genus *Camellia* (Table 1).

## Sampling methods

### Step description

**Figure 1.**
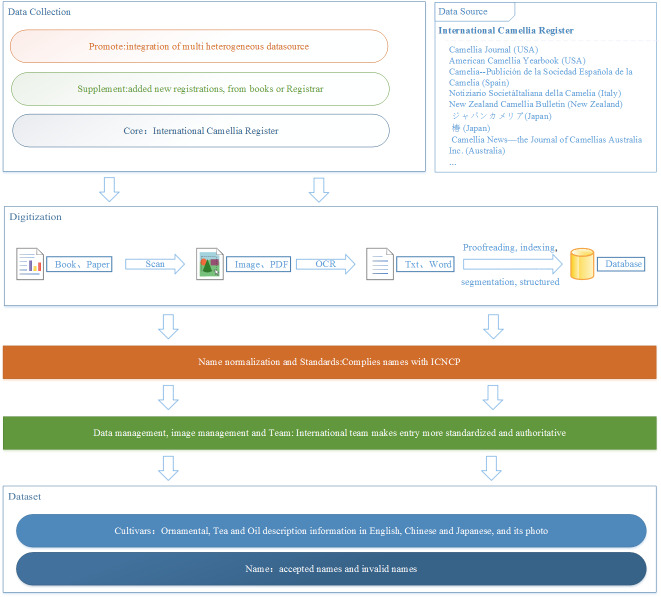
A flow chart of the dataset construction.

The camellia cultivar names were collected from books and journals, as well as new registrations through the International Camellia Registrar and Regional/Taxa Representatives responsible for registration from the year 1253 to 2019. These were then reviewed by experts on the online working platform, DICR. After treating important issues and problems existing within camellia names, the dataset was finally formed (Fig. 1).

**Data Source and Collection**

The dataset collected names of camellia cultivars from three aspects. The **core data** were from the printed books of the *International Camellia Register*, which contained all but the most recent of new camellia cultivars from all countries in the world. The **supplementary data** collected mainly from journals, such as the *International Camellia Journal* (ICS), *the Camellia Journal* (USA), *American Camellia Yearbook* (USA), *Camelia—Publicación de la Sociedad Española de la Camelia* (Spain), *Notiziario SocietàItaliana della Camelia* (Italy), *New Zealand Camellia Bulletin* (New Zealand), *ジャパンカメリア*（Japan Camellia), *椿* (Tsubaki, means camellia in Japanese), *Camellia News—the Journal of Camellias Australia Inc.* (Australia), *中国花卉园艺* (China Flowers & Horticulture) and some monographs (Japanese Camellia Society 2010, Sealy 1958, Macoboy 1981, Macoboy 1998, Takasi 1968, Japanese Camellia Society 1972, Gao et al. 2016). Gao’s latest book (Gao et al. 2016) records newly-released, mainly summer-flowering cultivars that can also bloom throughout the year. Finally, **new registrations** of the latest cultivars were collected through the International Camellia Registrar and those Regional/Taxa Representatives.

**Digitalisation**

The process of digitalisation had three main steps to digitalise the paper publications into structured entries, namely: scan process and optical character recognition (OCR) process, then structured process. The scan and OCR processes were somewhat easy, but the structured process of computer-readable text to entry was complex and laborious. Firstly, the computer-readable data required proofreading line by line. At the same time, the field name was manually indexed by reasoned design database structure, often manually. Then, the indexed record was automatically segmented to the structured entry by programming.

**Data management**

An International Team (http://camellia.iflora.cn/Home/Team), consisting mainly of the regional representatives for ornamental cultivars, as well as the taxa representatives for tea or oil cultivars, led by the International Camellia Registrar (Prof. Zhonglang Wang), were responsibile for managing cultivar information. This group ensured the authority and integrity of user-generated content in the online working platform.

To clarify the name relationship of cultivars, three taxonomic name levels were designed, namely: Accepted, Synonym and Unresolved. Each cultivar had one Accepted cultivar name and its coresponding Scientific name, Chinese name, as well as Japanese name. Some cultivars had many other names which were treated as Synonyms, based on the ICNCP. In addition, we collected English, Chinese and Japanese descriptions. The description basically followed those in the original publication, with some revisions to comply with the codes in ICNCP, such as adding the publications’ name, year and page,or changing the length unit from inches (USA) to centimetres (International Standard).

All names in the genus *Camellia* were treated to comply with the rules in ICNCP. Many problems were found when checking the historical names in the genus *Camellia*.

Three main problems were treated as follows:

1. The problem of re-use of epithets has caused many duplicate names.

According to the rules of Art. 30.1 and Art. 21.22 in ICNCP, the epithet of a cultivar must not be re-used within the same denomination class (as in the genus *Camellia*) for any other cultivars. Camellias with many duplicate names could cause confusion when transliterated or transcribed into Roman characters, especially Japanese cultivar names. For example, in the book *Camellias of Japan* (Japanese Camellia Society 2010) alone, there were 40 names (20 pairs) sharing the same names although they actually were completely different cultivars. Some of the names were different in Kanji, but when transliterated into Roman, they became indentical. Others were identical both in original Japanese and in Roman. This was a critical issue that needed immediate resolution.

In order to stabilise the names and to comply with the rules in ICNCP, we made some treatment using the following procedure: the first cultivar to bear a name was accepted, while for later named cultivars, the originator’s name, if known, was affixed in brackets, or different species or Group, different places of origin etc. affixed in brackets to distinguish them.

Within the four ICR books, there were 1982 duplicate names. Some names had more than two duplicate names. For example the name ‘Hagoromo’ had nine re-used names/duplicated names in ICR. In this dataset, we treated all existing duplicated names.

2. Names with the same Chinese characters, both in Chinese and in Japanese.

In Japanese cultivar names, there are many Chinese characters. These are called Kanji. Some are identical to Hanzi in Chinese, but pronounced differently. In ICNCP, there are some articles on how to deal with this issue. In future, when new cultivars are published, both in China and in Japan, the ICNCP instruction should be followed. According to Art. 21.23 of ICNCP, a name is not established if, on or after 1 January 1996, its cultivar epithet is (a) so similar in its original written form or (b) so similar or identical in pronunciation or (c) so similar or identical in spelling when transliterated or transcribed into the Roman alphabet (see Rec. 27F of ICNCP) to an existing epithet in the denomination class to which the cultivar is assigned, that the name might cause confusion.

For example, both in China and in Japan, there was a cultivar called 狮子头 in Chinese characters, but pronounced in different ways. It is pronouncied ‘Shizitou’ in China, while in Japan, it is pronounced ‘Shishigashira’. Although the Chinese characters were the same, they were actually different plants, so we treated them as two different cultivars by pronunciation in order to comply with ICNCP.

3. Diacritical Mark.

When we read rules at Art. 31.4 (7th edition in 2004) and at Rec. 34D.2 (8th edition in 2009 and 9th edition in 2016) of ICNCP: If a diacritical mark is used to indicate when a vowel is to be pronounced long in Romanised epithets transcribed from Kanji, Hiragana or Katakana, then the macron (overscore) is to be used and not the circumflex or any other diacritical mark. In Rec. 34D.2 Ex. 8, the epithet of Prunus ‘Chōshū-hizakura’ is not to be written as ‘Chôshû-hizakura’. However, many names in the genus *Camellia* use the circumflex ‘Chôshû-hizakura’ and many other forms, other than macron (overscore) ‘Chōshū-hizakura’. We changed all the circumflex to the macron (overscore) appearing in Romanised transcriptions of Japanese epithets, to comply with the Rules in ICNCP.

## Geographic coverage

### Description

**Figure 2.**
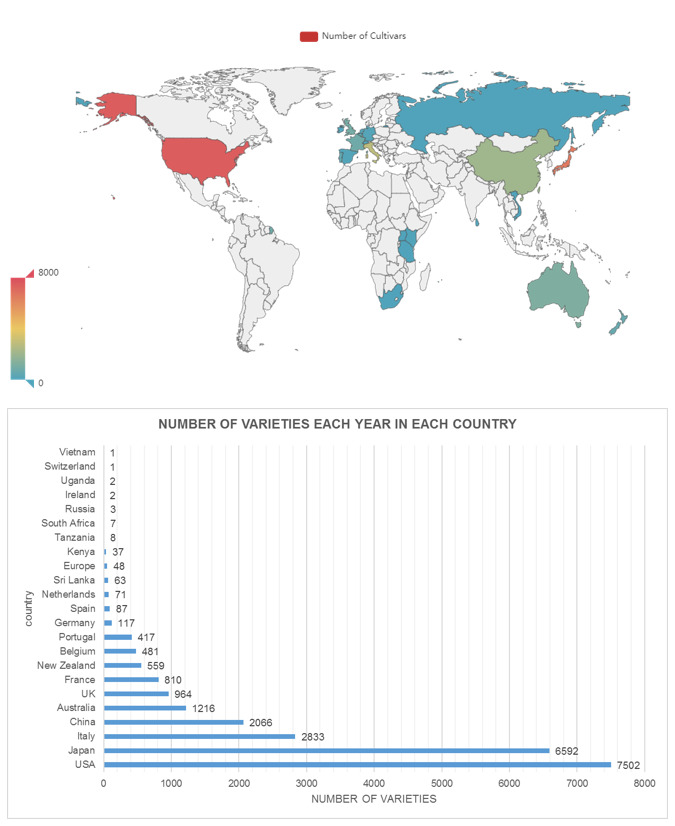
Map and number of cultivars in each country.

The geographic coverage contained 23 countries from the current data. The top five countries with most cultivars were USA (7,502), Japan (6,592), Italy (2,833), China (2,066) and Australia (1,216) (Fig. 2). The United States is rich in Camellia cultivars, due to many camellia enthusiasts and a convenient registration mechanism, which greatly promoted the development of cultivar breeding. Japan has a long history of camellia cultivation and has good records on cultivars with a wealth of books and illustrations. In European countries, a large amount of cultivars were bred between 1700 and 1900 and an abundance of books and illustrations on camellias were published in Italy. Although China has a very long history of tea, unfortunately many camellia cultivars have been lost. In recent decades, dozens of new cultivars, especially those with *C. azalea* as parents, have been produced and developed very quickly. The first ICS president, Prof. Waterhouse was Australian. Many other experts were also from Australia which helped develop strong Camellia interest in that country and hence helped Australia to be listed in the top five countries in the world.

## Taxonomic coverage

### Description

**Culitivars and its content**

**Figure 3.**
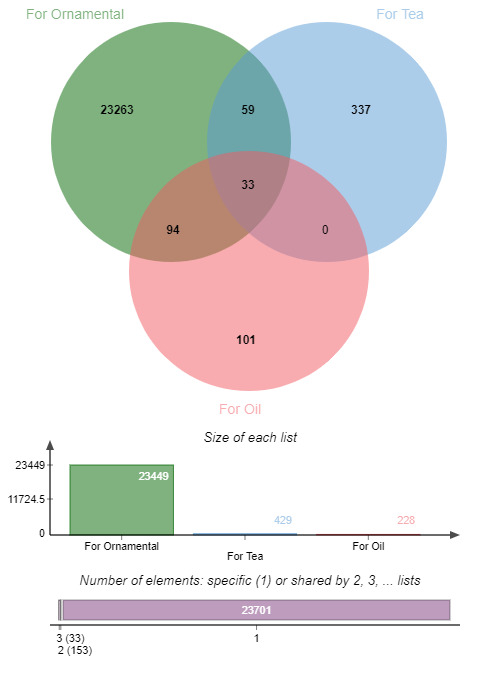
Venn diagram of economic value type for cultivars.

To date, in total, 45,210 names were published or released by different countries in the world. This included 23,887 accepted cultivar names and 21,323 other names (synonyms etc.), excluding 3,944 names which were believed extinct or no longer identifiable names. In total, 19,944 cultivar names are presently used. Now each cultivar had its scientific name, but so far, only 3932 cultivars had their Chinese names and 1040 cultivars had their Japanese names. All of them had English descriptions, while 3622 had Chinese descriptions and 973 had Japanese descriptions. Most of them were for ornamental use (23,449), 429 were for tea, 228 were for oil (Fig. 3). The Venn diagram below, made by jvenn software (Bardou et al. 2014), shows the intersectiing uses.

**Culitivar names and Synonyms**

This dataset recorded in total 21,323 synonyms, which also included orthographic error, erroneous synonym, Latin error, tentative designation, orthographic variant, corruption of the Japanese name, names in Chinese Hanzi, names in Japanese Kanji and Hiragana etc. Those synonyms reflected the history in which a cultivar was developed and introduced. Synonyms were also equally important compared to so-called valid names (accepted names). According to the ICNCP rules, accepted names are not fixed forever. When new evidence arises, some presently-accepted names can possibly become synonyms. For example, the very old cultivar name ‘Doncklaeri’ (1833) was a long-accepted name and was used widely in western countries for more than 100 years. New evidence shows that it was actually the same cultivar as the old Japanese cultivar ‘Masayoshi’ (1788). So, the name ‘Doncklaeri’ and its many variants (more than 20) have become synonyms for ‘Masayoshi’. This is also the reason why we need to include so-called invalid names and synonyms. Whether it is an accepted name or a synonym, it is not fixed. When more evidence is found, it enables accurate treatment for those names.

**Figure 4.**
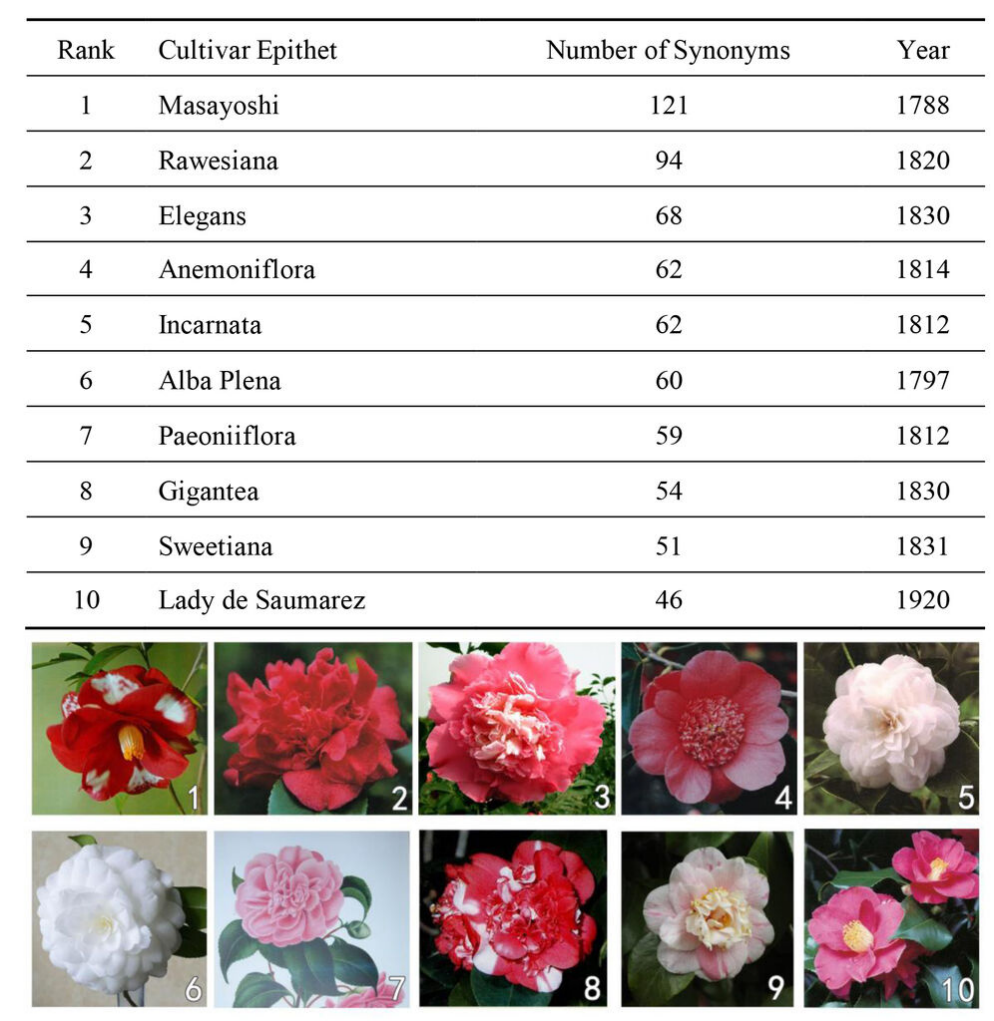
Cultivars with the largest number of synonyms.

From the present dataset, the cultivar with the largest number of synonyms was the above ‘Masayoshi’ which had 121 synonyms. The top ten cultivars with the most synonyms are listed in Fig. 4. This includes the number of synonyms, with the year released and its image. If you check these ten cultivars, you can see that all of them have beautiful names and are quite old. ‘Masayoshi’ for example, is very beautiful and was even designated as the logo flower for the 2010 International Camellia Congress held in Kurume, Japan in 2010. It was recorded that Dr von Siebold of the Dutch East India Company carried it to Europe in 1829 from his first journey to Japan. In 1883, he gave it another name - ‘Doncklaeri (Jorge 2014). When it was introduced to other countries, it was given many other names.

**Cultivars and their parentage**

**Figure 5.**
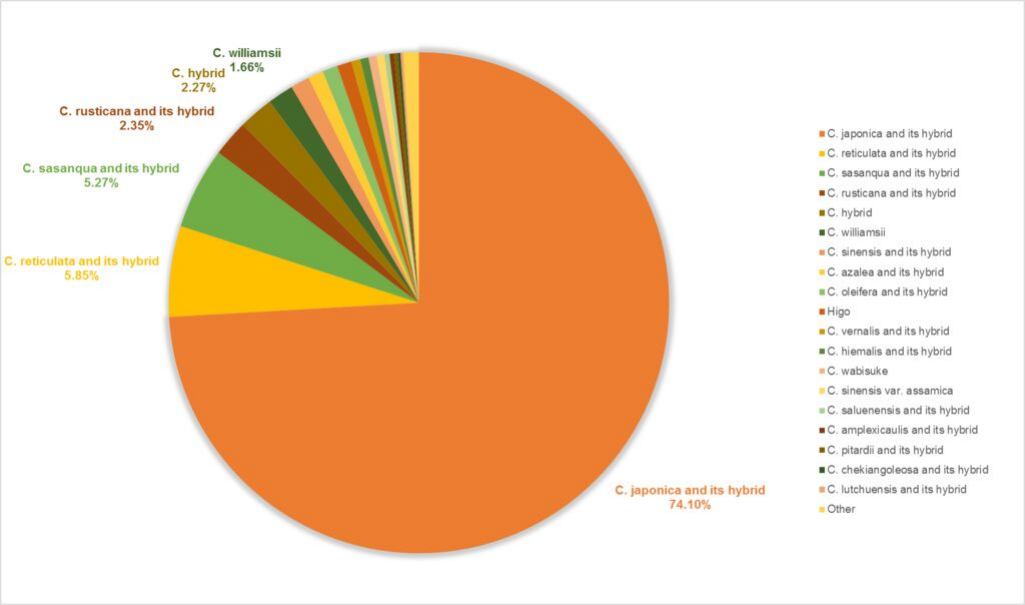
Pie chart of the species composition in horticultural use.

Fig. 5 shows the number of horticultural cultivars, *Camellia japonica* and its hybrids to be 18,141 or 74.10%, followed by those of *C. reticulata* and its hybrids (1,432 or 5.85%) and *C. sasanqua* and its hybrids (1,291 or 5.27%). It is generally agreed that the *C. japonica* arrived in London via the East India Company from China. The first plant of *C. japonica* was growing in England some time before 1739 in the greenhouse of Lord Petre. Since then, as it is very attractive and easy to grow and propagate, this speices has become the most popular of the ornamental camellias. Thousands of cultivars have been named. *Camellia reticulata* has a long history in Yunnan, China, but it was represented by only one plant in England in the 1800s. It did not hybridise with other camellias at that time and no other plants of *C. reticulata* were known in the western world, until the 1940s, when they were brought out from China. *Camellia reticulata* has the largest of all camellia flowers and has resulted in exquisite crosses with many other camellias. It has produced the second largest number of cultivars in the genus. *Camellia sasanqua* and its relatives are autumn blooming and native to southern Japan. Their leaves are generally smaller than those of *C. japonica.* The flowers of *C. sasanqua* shatter readily, making them unsuitable for cut flowers, but they make excellent landscape plants. Before the 1980s, *C. sansanqua* had the second largest number of camellia cultivars, but now it is ranked third.

## Temporal coverage

### Notes

**Figure 6.**
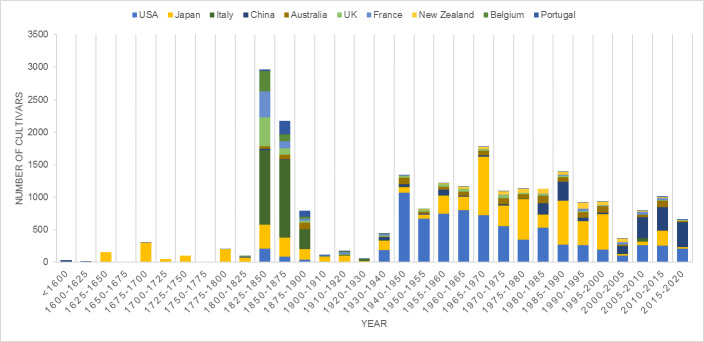
The number of cultivars produced each year in ten countries.

The data range of the dataset is 1253-2019. As seen from Fig. 6, the development history of camellia is closely related to the economic development of individual countries. Before 1600, there were only a few cultivars in the world. After introducing camellias to Western countries from China or Japan, the number of cultivars increased quickly. The first Industrial Revolution created wealth and more leisure time for some. Camellia breeding became popular and, in the middle of the 18th century, it reached the first peak of camellia cultivar numbers. From 1900-1945, the number of camellia cultivars decreased greatly as several wars happened in this perioid. After World War II, the number of camellia cultivars increased quickly and has been steadily growing for 70 years with the peak from 1965 to 1970. It can also be seen from the figures that China has a long history of Camellia cultivation. Since 2000, camellia development has escalated very quickly as prosperity in China greatly improved.

## Usage licence

### Usage licence

Creative Commons Public Domain Waiver (CC-Zero)

## Data resources

### Data package title

The dataset of Camellia cultivar names in the World

### Resource link

https://doi.org/10.5281/zenodo.4305197

### Number of data sets

2

### Data set 1.

#### Data set name

Cultivars

#### Data format

TSV file

#### Number of columns

18

#### Download URL

https://zenodo.org/record/4305197/files/Cultivars%20%28tsv%20for%20bak%29-The_Dataset_of_Camellia_Cultivar_Names_in_the_World.tsv?download=1

#### 

**Data set 1.** 

Column label	Column description
CultivarId	A unique number for each cultivar.
CultivarEpithet	The Cultivar Epithet for each cultivar.
ScientificName	The Scientific Name for each cultivar.
ChineseName	The Chinese Name for each cultivar.
JapaneseName	The Japanese Name for each cultivar.
Hiragana	The phonetic sounds in Japanese for each cultivar.
SpeciesOrCombination	Cultivar’s origin or cross parentage.
Meaning	The explanation of name.
CultivarType	The type of economic value, For Ornamental, For Tea Or For Oil.
DescriptionEn	The English Description for each cultivar.
DescriptionCn	The Chinese Description for each cultivar.
DescriptionJp	The Japanese Description for each cultivar.
YearPublished	The year of first publication.
Country	The country name which released the cultivar.
DefaultPhoto	The Type Image.
DefaultPhotoChosenBy	A specialist name who determined the Type image.
DefaultPhotoChosenDate	A date when the Type image was chosen by a specialist.
IsExtinct	Whether it was extinct or not.

### Data set 2.

#### Data set name

Synonyms

#### Data format

TSV file

#### Number of columns

4

#### Download URL

https://zenodo.org/record/4305197/files/Synonyms%20%28tsv%20for%20bak%29-The_Dataset_of_Camellia_Cultivar_Names_in_the_World.tsv?download=1

#### 

**Data set 2.** 

Column label	Column description
SynonymId	A unique number for each cultivar synonym.
Synonym	The synonym for each cultivar used.
Reference	The Reference recorded the synonym.
CultivarEpithet	The corresponding Cultivar Epithet for the synonym.

## Figures and Tables

**Table 1.** The Dataset Profile.

**Title**	The Dataset of Camellia Cultivars Names in the World
**Time range**	1253–2019
**Geographical scope**	World-wide, including 23 countries where Camellia Cultivars are popular
**Technology Type**	Data item extraction from books and journals, reviewed by experts
**Data format**	Submit through Excel, actually managed in MSSQL system
**Data service system**	Interantional Camellia Register's Website (http://camellia.iflora.cn)
